# Movement-Evoked Pain Versus Pain at Rest in Postsurgical Clinical Trials and Meta-Analyses: Protocol for a Follow-Up Systematic Review

**DOI:** 10.2196/15309

**Published:** 2020-01-22

**Authors:** Daenis Camiré, Jason Erb, Henrik Kehlet, Timothy Brennan, Ian Gilron

**Affiliations:** 1 Department of Anesthesiology and Perioperative Medicine Queen's University Kingston, ON Canada; 2 Section for Surgical Pathophysiology, Rigshospitalet Copenhagen University Copenhagen Denmark; 3 Department of Anesthesia University of Iowa Iowa City, IA United States

**Keywords:** postoperative pain, clinical trials, evoked pain, spontaneous pain, pain measurement

## Abstract

**Background:**

Postoperative pain is one of the most prevalent and disabling complications of surgery that is associated with personal suffering, delayed functional recovery, prolonged hospital stay, perioperative complications, and chronic postsurgical pain. Accumulating evidence has pointed to the important distinction between pain at rest (PAR) and movement-evoked pain (MEP) after surgery. In most studies including both measures, MEP has been shown to be substantially more severe than PAR. Furthermore, as MEP is commonly experienced during normal activities (eg, breathing, coughing, and walking), it has a greater adverse functional impact than PAR. In a previous systematic review conducted in 2011, only 39% of reviewed trials included MEP as a trial outcome and 52% failed to identify the pain outcome as either PAR or MEP. Given the recent observations of postsurgical pain trials that continue to neglect the distinction between PAR and MEP, this updated review seeks to evaluate the degree of progress in this area.

**Objective:**

This updated review will include postsurgical clinical trials and meta-analyses in which the primary outcome was early postoperative pain intensity. The primary outcome for this review is the reporting of MEP (vs PAR) as an outcome measure for each trial and meta-analysis. Secondary outcomes include whether trials and meta-analyses distinguished between PAR and MEP.

**Methods:**

To be consistent with the 2011 review that we are updating, this review will again focus on randomized controlled trials and meta-analyses, from Medical Literature Analysis and Retrieval System Online and EMBASE databases, focusing on pain treatment after thoracotomy, knee arthroplasty, and hysterectomy in humans. Trials and meta-analyses will be characterized as to whether or not they assessed PAR and MEP; whether their pain outcome acknowledged the distinction between PAR and MEP; and, for trials assessing MEP, which pain-evoking maneuver(s) were used.

**Results:**

Scoping review and pilot data extraction are under way, and the results are expected by March 2020.

**Conclusions:**

It is our belief that every postsurgical analgesic trial should include MEP as an outcome measure. The previous 2011 review was expected to have an impact on more widespread assessment of MEP in subsequent postoperative pain treatment trials. Thus, the purpose of this follow-up review is to reevaluate the frequency of use of MEP as a trial outcome, compared with PAR, in more recently published postoperative pain trials.

**Trial Registration:**

PROSPERO CRD42019125855; https://tinyurl.com/qw9dty8

**International Registered Report Identifier (IRRID):**

DERR1-10.2196/15309

## Introduction

### Background

Postoperative pain is one of the most prevalent and disabling complications of surgery that is associated with personal suffering, delayed functional recovery, prolonged hospital stay, perioperative complications, and chronic postsurgical pain [1]. Previous studies have distinguished between pain at rest (PAR) and movement-evoked pain (MEP) after surgery [2,3].

In most studies including both measures, MEP has been shown to be substantially more severe in intensity than PAR [4]. Furthermore, as MEP is commonly experienced during normal activities (eg, breathing, coughing, and walking), it has a greater adverse functional impact than PAR [5,6].

In 2011, a previous systematic review by Srikandarajah and Gilron showed that only 39% of reviewed trials included MEP as a trial outcome and 52% failed to identify the pain outcome as either PAR or MEP [4]. Consequently, an accompanying editorial by Kehlet and Dahl [7] confirmed that there has been no progress in the quality of assessment despite the need to include movement-associated pain in perioperative analgesic trials being emphasized almost 20 years previously [2]. Given the recent observations of postsurgical pain trials that continue to neglect the distinction between PAR and MEP, this updated review seeks to evaluate the degree of progress in this area. This previous systematic review focused on 3 surgical procedures—thoracotomy, knee arthroplasty, and hysterectomy—because postoperative analgesic clinical trials involving these procedures are relatively abundant in the literature and also because MEP is thought to be clinically relevant after these surgeries. To provide an appropriate estimate of change of the frequency of assessment of PAR versus MEP in postoperative analgesic trials, we chose to focus on these same 3 procedures in this review update.

### Objectives and Goal of This Study

The goals of this review are to evaluate postsurgical pain treatment trials for their ability to assess the frequency of use of PAR versus MEP as a trial outcome, the distinction between the 2, and what methods are used to assess MEP.

Main data to be extracted will be designation of MEP as the trial primary outcome, designation of PAR as the trial primary outcome, distinction between MEP and PAR in assessing pain, and method of evoking pain for the assessment of MEP.

## Methods

To be consistent with the 2011 review that we are updating, this review will, as much as possible, use the same methodology as used previously [4].

### Information Sources and Search Strategy

#### Clinical Trials

Analgesic clinical trials of pain after thoracotomy, knee arthroplasty, or hysterectomy will be searched on Medical Literature Analysis and Retrieval System Online and EMBASE databases (January 2014 to December 2019) as per a predefined search strategy (Multimedia Appendices 1-3). We chose a start date of 2014 so as to allow some time for the 2011 review to have been reflected in subsequent analgesic trials. Results, limited to randomized controlled treatment interventions involving humans, will be reviewed for inclusion. The search results from the 2 databases will then be combined for each surgical procedure, and any duplicates will be eliminated. For identified trials, articles will be eliminated if they are not randomized controlled trials (RCTs), involve a mix of surgeries, do not deal with results following surgery, did not measure pain scores, measured pain only after 1 week postoperatively, were not available in English, or could not be obtained. As the focus of this review is on pain outcome measurement only, the reviewed trials will not be evaluated with respect to trial quality or risk of bias.

#### Meta-Analyses

To search for meta-analyses specific to thoracotomy, knee arthroplasty, and hysterectomy, search strategies similar to those described in Multimedia Appendix 1 will be used, with the insertion of the term “meta-analysis” in place of the clinical trial search terms.

### Data Extraction and Analysis

The included trials will be characterized according to whether they measured MEP or not, whether they measured PAR or not, and whether the pain outcome(s) used in the trial acknowledged the distinction between MEP and PAR. We will also record, for each trial, which pain-evoking maneuver(s) were used (eg, coughing, walking, and joint flexion or extension) to facilitate the measurement of MEP. The included meta-analyses will be characterized according to whether they distinguished between PAR and MEP, whether they declared trials that failed to distinguish between PAR and MEP, and how they addressed the distinction (or lack thereof) between PAR and MEP across reviewed trials. Descriptive statistics will be used to synthesize extracted data. As the results from this review are not expected to be appropriate for statistical analysis, simple comparisons of the results of this review (ie, frequency of MEP measurement) with the results of the 2011 review will be made. For RCTs that measured MEP, the condition or maneuver associated with MEP assessment (eg, participant questioning about pain upon movement vs assessment of pain evoked by an investigator-witnessed standardized maneuver such as cough, force expiration, sitting from supine position, standing from seated position, and standardized walk test) will be tabulated across trials.

## Results

This review has been registered in the International Prospective Register of Systematic Reviews registry, CRD42019125855 [8]. Literature review and pilot data extraction are under way, and the results are expected by March 2020.

## Discussion

### Overview

Postoperative pain is one of the most prevalent and disabling complications of surgery. It can contribute to personal suffering, delayed functional recovery, prolonged hospital stay, and chronic postsurgical pain [1]. Over 50% of patients report moderate to severe pain in the early postoperative period, eg, postoperative day 0-3, and MEP is at least 200% more intense than PAR during this period [4]. Thus, it is critical to distinguish between MEP and PAR as the intensity of MEP is usually greater than that of PAR.

Mechanisms underlying MEP might be different from those underlying PAR [9,10]. In addition, MEP may respond differently to analgesic treatments than PAR [3]. Avoiding movement and activity by patients to minimize pain evoked by movement means that MEP can contribute to postoperative functional impairment [2].

Studies have shown [4] that MEP can be 95% to 226% more intense than PAR. As MEP is commonly experienced during normal activities (eg, breathing, coughing, and walking), it has a greater adverse impact on function and postoperative recovery than PAR. MEP affects the ability to relieve postoperative atelectasis, affects ambulation to improve blood flow, and reduces the risk of thromboembolism and other normal physical functions to promote musculoskeletal recovery, eg, after arthroplasty.

As stated previously, a previous systematic review by Srikandarajah and Gilron [4] showed that only 39% of reviewed trials included MEP as a trial outcome and 52% failed to identify the pain outcome as either PAR or MEP. In addition, 38% of reviewed trials did not specify the physical maneuver used to assess MEP, and 61% of trials did not capture the most severe pain condition. Furthermore, 5 out of the 7 (71%) meta-analyses did not distinguish between PAR and MEP.

### Conclusions

These gaps in methodology have implications with regard to trial precision, assessment, and treatment of pain during various activities. Different analgesics may have differential effects on PAR and during mobilization. It is important to distinguish between MEP and PAR and their response to novel treatment modalities when establishing a postsurgical analgesic trial. The assessment of pain should involve a standardized maneuver after a period of rest, ie, sitting from supine position. The maneuver for assessing MEP should be clinically relevant, ie, joint range of motion after arthroplasty.

It is our belief that every postsurgical analgesic trial should include MEP as an outcome measure. The previous 2011 review was expected to have an impact on the use of MEP as an important outcome measure in subsequent postoperative pain treatment trials. Thus, the purpose of this follow-up review is to evaluate the frequency of use of MEP as a trial outcome, compared with PAR, in more recently published postoperative pain trials.

## Appendix

Multimedia Appendix 1 Thoracotomy search strategy.

Multimedia Appendix 2 Arthroplasty search strategy.

Multimedia Appendix 3 Hysterectomy search strategy.

## Abbreviations

MEP: movement-evoked pain
PAR: pain at rest
RCT: randomized controlled trial
