# Supplementary material for: Movement-Evoked Pain Versus Pain at Rest in Postsurgical Clinical Trials and Meta-Analyses: Protocol for a Follow-Up Systematic Review
Source: JMIR Res Protoc. 2020 Jan 22;9(1):e15309. doi: 10.2196/15309 (PMC7003115; doi:10.2196/15309)
Supplement: Multimedia Appendix 1 [file resprot_v9i1e15309_app1.docx]

**Appendix 1: Thoracotomy search strategy**

**Medline**

**1 exp Thoracotomy/ (6072)**

**2 exp Pneumonectomy/ (17114)**

**3 exp Pulmonary Surgical Procedures/ (44764)**

**4 exp Pain! (237217)**

**5 exp Analgesia/ (25943)**

**6 pain$.mp. (358187)**

**7 1 or 3 or 2 (49960)**

**8 6 or 4 or 5 (432653)**

**9 8 and 7 (1708)**

**10 limit 9 to (humans and randomized controlled trial) (267)**

**Embase**

**1 exp Thoracotomy/ (10630)**

**2 exp Lung Resection! (8503)**

**3 exp Lung Surgery/ (24049)**

**4 exp Pain! (362074)**

**5 exp Analgesia/ (59140)**

**6 pain$.mp. (359586)**

**7 1 or 3 or 2 (33043)**

**8 6 or 4 or 5 (493621)**

**9 8 and 7 (2730)**

**10 limit 9 to (human and "treatment (2 or more terms min difference)") (395)**
