# Supplementary material for: Movement-Evoked Pain Versus Pain at Rest in Postsurgical Clinical Trials and Meta-Analyses: Protocol for a Follow-Up Systematic Review
Source: JMIR Res Protoc. 2020 Jan 22;9(1):e15309. doi: 10.2196/15309 (PMC7003115; doi:10.2196/15309)
Supplement: Multimedia Appendix 2 [file resprot_v9i1e15309_app2.docx]

**Appendix 2: Arthroplasty search strategy**

**Medline**

**1 exp Knee Prosthesis/ (6625)**

**2 exp Arthroplasty, Replacement, Knee/ (6994)**

**3 1 or 2 (11807)**

**4 exp Pain/ (240667)**

**5 exp Analgesia/ (26276)**

**6 pain$.mp. (367771)**

**7 6 or 4 or 5 (442699)**

**8 3 and 7 (2331)**

**9 limit 8 to (humans and randomized controlled trial) (333)**

**Embase**

**1 exp Knee Prosthesis/ (10693)**

**2 exp Arthroplasty, Replacement, Knee/ (22985)**

**3 1 or 2 (25880)**

**4 exp Pain/ (300932)**

**5 exp Analgesia/ (72610)**

**6 pain$.mp. (443740)**

**7 6 or 4 or 5 (511550)**

**8 3 and 7 (6087)**

**9 limit 8 to (human and “treatment (2 or more terms min difference)” and article) (571)**
