# Supplementary material for: Movement-Evoked Pain Versus Pain at Rest in Postsurgical Clinical Trials and Meta-Analyses: Protocol for a Follow-Up Systematic Review
Source: JMIR Res Protoc. 2020 Jan 22;9(1):e15309. doi: 10.2196/15309 (PMC7003115; doi:10.2196/15309)
Supplement: Multimedia Appendix 3 [file resprot_v9i1e15309_app3.docx]

**Appendix 3: Hysterectomy search strategy**

**Medline**

**1 exp Hysterectomy/ (20086)**

**2 exp Pain! (225760)**

**3 exp Analgesia/ (24968)**

**4 pain$.mp. [mp=title, original title, abstract, name of substance word, subject heading word] (339113)**

**5 2 or 3 or 4 (410764)**

**6 1 and 5 (1487)**

**7 limit 6 to (humans and randomized controlled trial) (357)**

**8 from 7 keep 1-357 (357)**

**Embase**

**1 exp HYSTERECTOMY/ (18504)**

**2 exp PAIN/ (343296)**

**3 exp ANALGESIA! (57238)**

**4 pain$.mp. [mp=title, abstract, subject headings, heading word, drug trade name, original title, device manufacturer, drug manufacturer name] (342608)**

**5 2 or 3 or 4 (471084)**

**6 1 and 5 (2745)**

**7 limit 6 to (human and female and "treatment (2 or more terms high specificity)") (276)**

**8 limit 6 to (human and female and "treatment (2 or more terms high sensitivity)") (986)**

**9 limit 6 to (human and female and "treatment (2 or more terms min difference)") (453)**

**10 from 9 keep 1-453 (453)**
